# Within Host Evolution Selects for a Dominant Genotype of *Mycobacterium tuberculosis* while T Cells Increase Pathogen Genetic Diversity

**DOI:** 10.1371/journal.ppat.1006111

**Published:** 2016-12-14

**Authors:** Richard Copin, Xueying Wang, Eddie Louie, Vincent Escuyer, Mireia Coscolla, Sebastien Gagneux, Guy H. Palmer, Joel D. Ernst

**Affiliations:** 1 Division of Infectious Diseases, Department of Medicine, New York University School of Medicine, New York, NY, United States of America; 2 Department of Mathematics and Statistics, Washington State University, Pullman, WA, United States of America; 3 Microbiology laboratory, Wadsworth Center, New York State Department of Health, Albany, NY, United States of America; 4 Department of Medical Parasitology and Infection Biology, Swiss Tropical and Public Health Institute, Basel, Switzerland; 5 University of Basel, Basel, Switzerland; 6 Paul G. Allen School for Global Animal Health, Washington State University, Pullman, WA, United States of America; 7 Department of Microbiology, New York University School of Medicine, New York, NY, United States of America; 8 Department of Pathology, New York University School of Medicine, New York, NY, United States of America; University of Washington, UNITED STATES

## Abstract

Molecular epidemiological assessments, drug treatment optimization, and development of immunological interventions all depend on understanding pathogen adaptation and genetic variation, which differ for specific pathogens. *Mycobacterium tuberculosis* is an exceptionally successful human pathogen, yet beyond knowledge that this bacterium has low overall genomic variation but acquires drug resistance mutations, little is known of the factors that drive its population genomic characteristics. Here, we compared the genetic diversity of the bacteria that established infection to the bacterial populations obtained from infected tissues during murine *M*. *tuberculosis* pulmonary infection and human disseminated *M*. *bovis* BCG infection. We found that new mutations accumulate during *in vitro* culture, but that *in vivo*, purifying selection against new mutations dominates, indicating that *M*. *tuberculosis* follows a dominant lineage model of evolution. Comparing bacterial populations passaged in T cell-deficient and immunocompetent mice, we found that the presence of T cells is associated with an increase in the diversity of the *M*. *tuberculosis* genome. Together, our findings put *M*. *tuberculosis* genetic evolution in a new perspective and clarify the impact of T cells on sequence diversity of *M*. *tuberculosis*.

## Author Summary

*Mycobacterium tuberculosis* is amongst the most successful and enigmatic pathogens that has burdened humanity for thousands of years. The success of this pathogen depends on unique strategies employed to adapt during infection. Understanding these strategies is key to decipher the complexity of *M*. *tuberculosis* and crucial for epidemiological predictions and drug treatments. However, little is known on the impact of transmission population bottlenecks and host immune pressures on sequence diversity of mycobacteria. By combining deep sequencing and parallel evolution, we characterized the evolution of *M*. *tuberculosis* genetic diversity during the course of pulmonary infection in mice, and in disseminated *M*. *bovis* BCG infection in humans. We found that under *in vitro* rich culture conditions, multiple adaptive mutations arise, but none of these generate lasting allele diversity *in vivo*. We found that this phenomenon was not uniquely dictated by transmission bottleneck and that within host evolution contribute to select for a dominant genotype during *M*. *tuberculosis* infection. Finally, we used a immuno-deficient mouse model to show that T cells are contributor rather than limitator of genetic diversity during infection. Together, our results emphasize the complexity and uniqueness of *M*. *tuberculosis* and contribute understanding *M*. *tuberculosis* genetic adaptation.

## Introduction

Microbial pathogens adapt to host environments to establish replicative niches and counter immune responses. Adaption, which commonly relies on the generation and transmission of genetic variants with increased fitness, is especially critical for obligate pathogens that must infect, replicate, and be transmitted to new hosts to survive and propagate. While mutations emerge randomly in the genome, the accumulation and loss of variants is the result of both genetic drift and natural selection, and is influenced by transmission population bottlenecks within and between hosts. The large reductions in population size during bottlenecks can have strong evolutionary effects, leading to erosion of genetic diversity and reduction in evolutionary potential and individual fitness through random fixation of slightly deleterious alleles[1, 2]. The severe nature of bottlenecks implies that the success of infection depends on 1) the genetic diversity of the pathogen population in the donor host and, 2) the selection of adapted genotypes during transmission. For HIV and other RNA viruses, founder particles are biased to favor the transmission of variants associated with increased fitness[3, 4]. The corresponding increase in fitness results from the selection and contribution of low frequency variants, and not to the effect of a single, dominant genome. The same principle applies also to other pathogens, including certain bacteria and parasites[5] [6].

Understanding within-host pathogen population diversity has important implications for drug treatment and resistance[7]^,^[8], and for inferring transmission networks[9]^,^[10]^,^[11] and evolutionary processes[12]. Two opposing models of within-host microbial evolution have been described[6]: a ‘dominant-lineage’ model, in which beneficial mutations lead to unique genotypes to establish and maintain infection, and a ‘diverse-community’ model, in which minor variants rise to intermediate frequencies and coexist with major variants in the microbial population. Here, we distinguish between these models and determine the roles of drift, bottlenecks, and selection in genetic variation of *Mycobacterium tuberculosis* during infection.

Members of the *M*. *tuberculosis* complex (MTBC) cause tuberculosis, a chronic infection transmitted by aerosol that remains a deadly disease, despite the availability of drug treatment[13]. *M*. *tuberculosis* is an obligate pathogen, and has no natural ecological niche other than its human hosts, with which it has coevolved for thousands of years[14, 15]. Immunological control of *M*. *tuberculosis* depends on T lymphocytes, which recognize peptide fragments of bacterial proteins bound to polymorphic MHC (HLA in humans) molecules[16]. The MTBC is characterized by a largely clonal population structure classified into 7 main human-adapted phylogenetic lineages[15]^,^[17]. Despite thousands of years after divergence from a common ancestor, all MTBC lineages share identical 16S rRNA sequences and 99.9% nucleotide identity at the whole genome level[18]. However, *M*. *tuberculosis* can generate diversity over short and long time courses. For example, most drug resistance determinants in the MTBC represent chromosomal mutations selected by drug exposure[18]. Moreover, population genetic analyses have highlighted that nonsynonymous single nucleotide polymorphisms (nSNPs) tend to accumulate in the *M*. *tuberculosis* genome at a higher rate than in related organisms[19]^,^[17]. Because nSNPs are often deleterious, it was suggested that *M*. *tuberculosis* undergoes random genetic drift associated with serial population bottlenecks[17]. Yet, *M*. *tuberculosis* does not show the typical signs associated with increased genetic drift[18]. Indeed, its genome has moderate numbers of insertion sequences, few pseudogenes, and no obvious other signals of extensive genome degradation.

Several findings support the notion that within-host selection plays a critical role in shaping the genome of the MTBC. Comparison of a globally representative sample of *M*. *tuberculosis* isolates yielded evidence of strong purifying selection, with different patterns of selection related to gene function[20]. We also found that antigen conservation dominates in this pathogen, and that the vast majority of the currently known T cell epitopes are more conserved than any other part of the genome, indicating that these sequences are under strong selective pressure[21]. Although the factors driving epitope conservation in *M*. *tuberculosis* are still undetermined, these observations led to the hypothesis that human T cell recognition could play an important role by limiting genetic diversity[21]. In the context of drug exposure, although within-host selection is a source of heterogeneity, the frequency of mutations is low and their locations in the genome highly specific[22].

To better understand conservation and diversity of *M*. *tuberculosis* during infection, and to delineate the contribution of T cell selection, we combined high-density whole-genome sequencing (WGS) and mathematical modeling to assess the evolution of genetic diversity of *M*. *tuberculosis* in murine pulmonary infection, and in human disseminated *M*. *bovis* BCG infection.

## Results

### Genetic diversity in a H37Rv stock targets key metabolic proteins

In the widely-studied virulent strain of *M*. *tuberculosis*, H37Rv, genomic differences among stocks from individual laboratories have been reported, indicating that *in vitro* culture generates *M*. *tuberculosis* diversity[23]. To further understand this observation, we sequenced our laboratory stock and compared it to that of the H37Rv reference (NC_000962)[24]. Deep sequencing revealed that the stock population was heterogeneous and contained 34 polymorphisms present at various frequencies (S1A Table). Among those mutations, 25 were nonsynonymous single nucleotide changes affecting 25 proteins (Table 1); nonsynonymous mutations were overrepresented in genes whose products are involved in metabolic pathways with potentially important in vivo functions (observed = 12, expected = 7; χ^2^, *p<0*.*05*; Table 1). Notably, we observed that 28% of the bacterial population had a nSNP in the gene encoding Isocitrate lyase 1 (Icl1), an enzyme essential for allowing net carbon gain by diverting acetyl-CoA from β-oxidation of fatty acids into the glyoxylate shunt pathway[25]^,^[26]. Disruption of *icl1* attenuates *M*. *tuberculosis* persistence and virulence in mice without affecting bacterial growth during the acute phase of infection. An additional metabolic gene, *kgd*, contained an nSNP in 28% of our stock and encodes an α-ketoglutarate decarboxylase involved in an alternative pathway that generates succinate for the tricarboxylic acid cycle, which may help the pathogen cope with hypoxia[27]. Since Icl1 and Kgd are employed in oxygen- or glucose-deprived environments, we hypothesized that they do not play a key role during growth in rich culture media. This hypothesis was supported by the predicted impact of the identified nSNPs on protein function and/or stability: amino acid substitutions in 17 (68%) of the 25 mutated proteins, including Icl1 and Kgd, were predicted to be deleterious (Table 1). Taken together, these data indicate that during in vitro growth, MTBC accumulates mutations in genes which are not relevant for in vitro growth but might be key for in vivo growth and/or transmission.

**Table 1:** Characteristics of nSNP-containing genes identified in initial *M*. *tuberculosis* inoculum population.

*Mutation nature*	*Locus tag*	*Protein name*	*Amino acid substitution*	*Putative function*	*Functional Category*	*Amino acid change tolerance*
nSNP	Rv0282	EccA3	V482G	ESX-3 secretion system protein	Transport	Deleterious
nSNP	Rv0422c	ThiD	Q21K	Thiamine metabolism	Metabolism	Deleterious
nSNP	Rv0467	Icl1	A148G	Glyoxylate - dicarboxylate metabolism	Metabolism	Deleterious
nSNP	Rv0587	YrbE2A	V146G	ABC - phospholipid transporters	Transport	Deleterious
nSNP	Rv0969	CtpV	A490P	Cation-transporting ATPase	Transport	Deleterious
nSNP	Rv1248c	Kgd	T675P	Alternate tricarboxylic acid cycle	Metabolism	Deleterious
nSNP	Rv1307	AtpH	V241G	Oxidative phosphorylation	Metabolism	Deleterious
nSNP	Rv1384	CarB	A860G	Pyrimidine metabolism - alanine, aspartate - glutamate metabolism	Metabolism	Deleterious
nSNP	Rv1661	Pks7	R808P	Lipid biosynthesis proteins - polyketide synthase	Metabolism	Deleterious
nSNP	Rv1666c	Cyp139	V1G	Bisphenol, aminobenzoate, limonene and pinene degradation	Metabolism	Neutral
nSNP	Rv1695	PpnK	V294G	Nicotinate - nicotinamide metabolism	Metabolism	Deleterious
nSNP	Rv1956	HigA	V96G	Antitoxin	Detoxification	Neutral
nSNP	Rv2143	Hypothetical protein	H106P	Putative phosphoribosyl transferase	Metabolism	Neutral
nSNP	Rv2290	LppO	Y125S	Lipoprotein	Cell wall	Deleterious
nSNP	Rv2395	Hypothetical protein	S625P	Unknown	Transport	Deleterious
nSNP	Rv2682c	Dxs1	A424P	Thiamine metabolism - terpenoid biosynthesis	Metabolism	Neutral
nSNP	Rv2724c	FadE20	N245T	Oxidoreductases - geraniol degradation	Metabolism	Deleterious
nSNP	Rv2809	Hypothetical protein	N57T	Unknown	Unknown	Deleterious
nSNP	Rv3091	Hypothetical protein	H217P	Unknown	Unknown	Neutral
nSNP	Rv3270	CtpC	A166E	Manganese/zinc-exporting P-type ATPase	Transport	Deleterious
nSNP	Rv3313c	Add	S325G	Purine metabolism	Metabolism	Neutral
nSNP	Rv3584	LpqE	V95G	Lipoprotein	Cell wall	Deleterious
nSNP	Rv3691	Hypothetical protein	A62G	Unknown	Unknown	Neutral
nSNP	Rv2157c*	murF	L399V	Lysine biosynthesis - peptidoglycan biosynthesis - vancomycin resistance	Cell wall	Deleterious
nSNP	Rv0272c*	Hypothetical protein	H91R	Putative serine aminopeptidase	Unknown	Neutral

### Genomic selection in M. tuberculosis during serial passage in immunocompetent or T cell deficient mice

**Fig 1:**
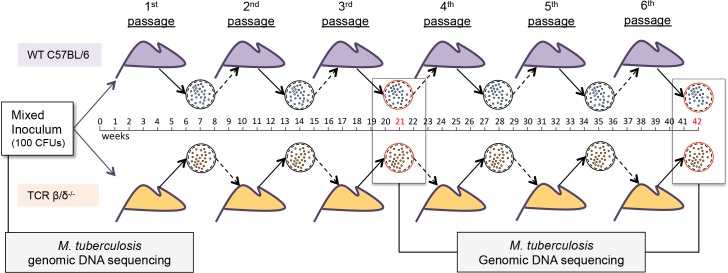
Mouse passage protocol.

Based on our observation that T cell epitopes are the most conserved regions of the MTBC genome, we tested whether T cell immunity constrains genetic diversity during infection. We used WGS to compare the frequency of variants in *M*. *tuberculosis* after six in vivo passages of the H37Rv stock containing sequence variants in immunocompetent wild-type (WT) or in T cell-deficient (TCR β/δ^-/-^) mice (Fig 1). Following the third and sixth passages, a pool of *M*. *tuberculosis* bacteria from the lungs of each mouse was sequenced and compared to the sequence of the initial aerosol inoculum.

5 Wild type (C57BL/6; purple) or 5 T cell-deficient (TCR β/δ^-/-^; orange) mice were each infected with 100 CFU of *M*. *tuberculosis* H37Rv stock. After 6 weeks of infection, the lungs of each mouse were homogenized to prepare an inoculum used to infect the next set of mice of the same phenotype (see Methods). Genomic DNA samples of the initial inoculum and of the bacterial populations after the 3^rd^ and 6^th^ mouse passages were examined by WGS.

### In vivo selection in mice promotes reversion towards consensus sequence of M. tuberculosis

**Fig 2:**
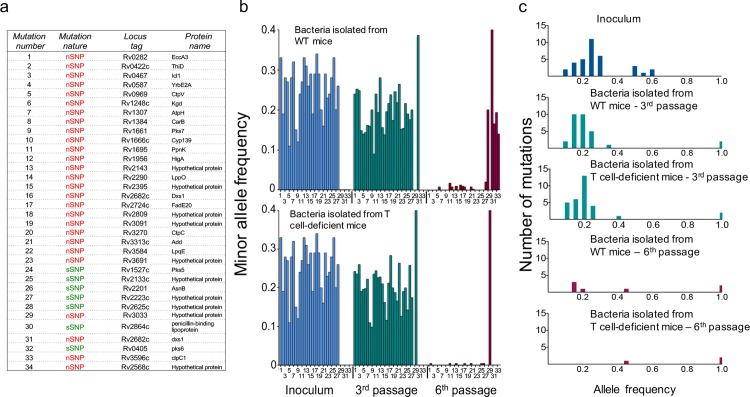
Sequence variants present in the initial inoculum are purified out during mouse passages. (a) List of SNPs present in the initial inoculum population and the mouse isolates at a frequency <50%. (b) Evolution of SNPs found in the initial inoculum (blue), following the third (green) and sixth (red) mouse passages in WT and T cell deficient mice. Mutation numbers (from the list in panel a) are indicated on the x axis. (c) Number of variant alleles with high, intermediate, and low frequencies identified in the initial *M*. *tuberculosis* inoculum (blue) and in the *M*. *tuberculosis* populations isolated after the third (green) and sixth (red) mouse passages.

*In vivo* passaging allowed 14 and 18 doublings of the bacteria in lungs of WT and T cell deficient mice, respectively, followed by a ~10,000-fold bottleneck during infection of the next group of mice, so we were able to characterize the impact of transmission bottlenecks and *in vivo* selection on the genetic diversity within the inoculum population. We monitored the frequencies of the 34 pre-existing polymorphisms in the *M*. *tuberculosis* population after the third and sixth passages in the two groups of mice. Following three passages, 4 variants were purified out, and 2 achieved fixation in the isolates from both immunocompetent and T cell-deficient mice. These 6 variants were present at the highest frequency (≥50%) in the initial inoculum population (S1A Table) and the majority (4/6) were sSNPs or intergenic mutations. In contrast, 82% (23/28) of the minor variants present in the initial inoculum and that were maintained after three passages were nSNPs (Fig 2A). The frequency of these 28 variants was decreased compared to that in the inoculum population (average total frequencies: 25% in the inoculum,19% in the WT mouse isolates and 20% in the T cell deficient mouse isolates; multiple t test of coverage, *p<0*.*01*; Fig 2). These observations indicated that mixed populations of *M*. *tuberculosis* continued to exist in both groups of mice after three passages. However, after six passages, all of the minor variants (28/28) were lost (polymorphic reads <5%) in both mouse groups and each mouse was infected with a clonal *M*. *tuberculosis* population closer to the H37Rv reference sequence (Fig 2). To infer whether some variants identified in the initial inoculum might be linked, we analyzed the changes in frequencies of each SNP after the three first passages in WT and T cell-deficient mice. We found 5 sets of variants containing 2 to 3 SNPs each that evolved in parallel (multiple t test of coverage, *p<0*.*05*) during in vivo passage in both mouse groups, suggesting that these mutations were linked (S2 Table).

### Mathematical modeling shows that in vivo selection of M. tuberculosis follows a dominant lineage model of evolution independent of T cells

**Fig 3:**
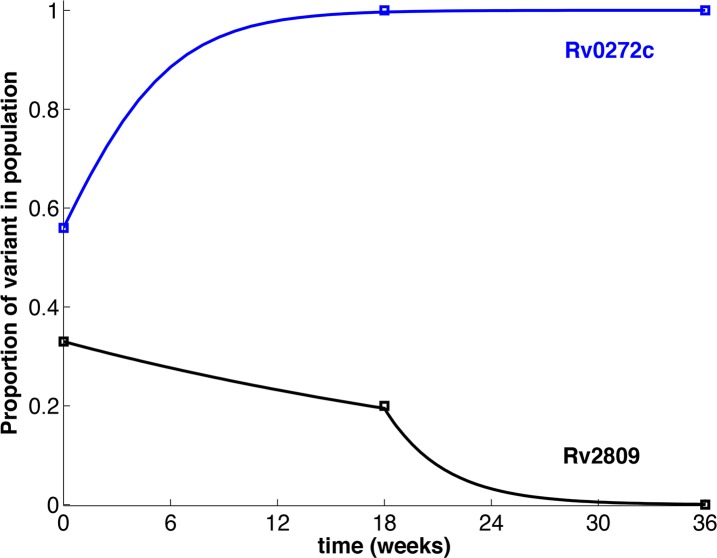
Fixation of variant alleles in the bacterial population progresses by a deterministic process. The squares indicate the proportion of variants in the inoculum and at specific recovery timepoints following serial infection of mice. The curves represent the modelled progression as influenced by a decision marker. Rv0272c (blue line) represents a variant that undergoes rapid purification leading to fixation after 3 passages; Rv2809 (black line) represents two-stage purification leading to fixation after 6 passages. Rapid purification resulting in either full or zero fixation within 3 passages occurred for all variant alleles present at ≥ 50% in the original inoculum; two-stage fixation occurred for all variant alleles present at <50% in the inoculum (S1 Table).

The data are not consistent with a simple dilutional bottleneck, since minor sequence variants were not progressively lost from the population. Instead, the frequency of the minor variants was unchanged or only slightly reduced after the third passage, but were absent after the sixth passage (S1 Table). In contrast, the sequence variants that were present as a higher fraction of the initial inoculum were purified within the first 3 passages, resulting in fixation or loss (S1 Table). This pattern was independent of the presence of T cells. To explain the observed purification dynamics, we developed a mathematical model using a system of ordinary differential equations, analyzed by dynamical system theory[28]. The model parameters were estimated from the data by the generalized profiling method[29]. The model results strongly supported that *in vivo* selection of *M*. *tuberculosis* populations is driven by a deterministic process for which the kinetics depend on the frequencies of variants in the initial inoculum population (Fig 3). When the variant allele frequency in the inoculum population was less than 50%, the purifying process consisted of two steps: the first step was characterized by the competitive co-existence of minor genotypes. This co-existence continued for a minimum of three passages. The second step was a dynamic process, occurring during or after passage 4, resulting in fixation of polymorphisms and purification of a single *M*. *tuberculosis* population. In contrast, when the percentage of variants in the inoculum was equal to or greater than 50%, the bacterial population underwent a rapid purification process that occurred within the first 3 passages, leading to either elimination of these mutants or their fixation as the dominant genotype. These results show evidence that 1) *M*. *tuberculosis* evolution is deterministic and follows a “dominant lineage” model *in vivo* and 2) is a dynamic process largely independent of the presence of T cells that cannot be explained by passive transmission bottlenecks.

### T cells increase the diversity of M. tuberculosis during prolonged infection

To determine whether within-host selection at specific loci can also generate variation, and to examine the contribution of T cells in generating this diversity, we characterized the polymorphisms that appeared *de novo* in the *M*. *tuberculosis* population after infection of wild-type and T cell-deficient mice. Genome sequencing of bacterial populations following the third mouse passages revealed 2 minor variants that were undetectable in the initial inoculum and present in the WT (1 mutation) or T cell deficient mouse isolates (1 mutation) (Table 2 and S1B Table). While no additional polymorphisms were detected in the T cell deficient isolates after the 6^th^ passage, 4 mutations appeared in the genomes of the WT mouse isolates. Among the polymorphisms identified in WT mouse isolates, 4 were nSNPs (Table 2). By contrast, the unique mutation identified in the bacterial populations from T cell deficient mice was a synonymous SNP (Table 2). Together, these results indicate that T cell-dependent immunity can contribute to sequence diversity in *M*. *tuberculosis*.

**Table 2:** Characteristics of mutated genes identified in the *M*. *tuberculosis* mouse lung populations.

*Mouse group*	*Mutation nature*	*Locus tag*	*Protein name*	*Amino acid substitution*	*Putative function*	*Functional category*	*Amino acid change tolerance*
WT	nSNP	Rv2682c	Dxs1	R163L	Thiamine metabolism & Terpenoid biosynthesis	Metabolism	Deleterious
WT	nSNP	Rv3596c	ClpC1	K186E	Regulatory ATPase	Metabolism	Deleterious
WT	nSNP	Rv3033	Hypothetical protein	V42A	Unknown	Unknown	Neutral
WT	nSNP	Rv2568c	Hypothetical protein	A313S	Unknown	Unknown	Neutral
WT	sSNP	Rv0405	Pks6	-	Membrane bound polyketide synthase	Metabolism	-
T cell deficient	sSNP	Rv2864c	PBL	-	Penicillin-binding lipoprotein	Cell wall and cell processes	-

To further evaluate the impact of T cells on the genome diversity of *M*. *tuberculosis* during infection, we determined the average mutation rates of *M*. *tuberculosis* in both mouse groups. Considering a similar bacterial generation time of 20 hours for both groups, we estimated that the average mutation rate of *M*. *tuberculosis* was highly reduced during prolonged infection in T cell deficient mice (3.8 x 10^-9^ in WT mice versus 7.7 x 10^-10^ in T cell deficient mice) and this, despite a higher bacterial burden in T cell deficient mice (2 logarithms). Thus, the reduction in the mutation rate calculated for the T cell deficient mouse isolates strongly supports a role for T cells in generating diversity in *M*. *tuberculosis* during prolonged infection.

### Genetic diversity of *M*. *bovis* BCG in disseminated human infection support a dominant lineage model of evolution

**Fig 4:**
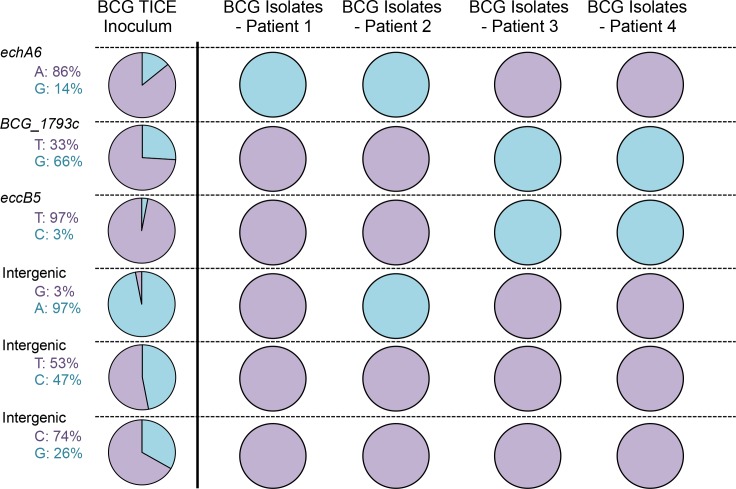
Frequencies of SNPs identified in *M*. *bovis* BCG inoculum and in the disseminated *M*. *bovis* BCG isolated from patients treated for bladder cancer. Frequencies of variants are indicated and represented in the pies in light blue. Sequences corresponding to the published sequence of *M*. *bovis* BCG Tice are indicated and represented in purple.

To determine whether the results obtained in mice are also relevant in human infections, we examined the extent and nature of within-host selection of slow-growing mycobacteria isolated from human patients. Since it is not possible to determine the sequence of the inhaled inoculum that establishes human tuberculosis, we took an alternative approach. Invasive carcinoma of the urinary bladder in immunocompetent patients is commonly treated by instillation of a standardized preparation of mycobacteria (*M*. *bovis* BCG). In rare cases, disseminated BCG infection develops, and involves tissues beyond the bladder[30]. Since the BCG inoculum is defined and prepared according to pharmaceutical standards, we could be confident in the identity of the bacteria to which a patient was exposed. We first determined the genome sequence of the inoculum used to treat bladder cancer in the United States, *M*. *bovis* BCG Tice. Similarly to what we observed for our H37Rv inoculum, the BCG Tice inoculum population was heterogeneous and contained 6 variants present at frequencies from 3% to 66% (S3 Table and Fig 4). Two of these variants were nSNPs present in *echA6* or *eccB5*. The gene *echA6* encodes a putative enoyl-CoA hydratase capable of supplying energy and carbon from fatty acid β-oxidation during starvation[31]. EccB5 is a membrane protein of the ESX-5 type VII secretion system involved in secretion of proteins and uptake of nutrients[32]. Both proteins are thus implicated in metabolic pathways used in potentially hostile environments.

To characterize the evolution of these minor variants during dissemination in humans, we sequenced *M*. *bovis* BCG isolates from 4 patients. We found that the heterogeneity of the inoculum population was eliminated in vivo, as the four variants were either purified out or achieved fixation (Fig 4). Together, these results reinforce the conclusion that *M*. *tuberculosis* populations follow a dominant lineage model of evolution during infection.

## Discussion

The combination of genomics and mathematical modeling to determine the impact of *in vivo* evolution on sequence diversity of *M*. *tuberculosis* yielded evidence that conservation of the *M*. *tuberculosis* genome is driven by a deterministic process leading to the selection of a dominant genotype in vivo. By examining evolution of a heterogeneous population of *M*. *tuberculosis* from *in vitro* passaged bacteria during prolonged infection in mice, we found that conservation of *M*. *tuberculosis in vivo* is driven by selection forces limiting genome diversity acquired *in vitro*. Even though the variants in the initial inoculum population could replicate *in vivo*, within-host selection limited their ability to compete with the rest of the population, and a single dominant genome eventually emerged. These results indicate that specific mutants are purged in vivo and that a dominant lineage establishes and maintains long–term infection. This has important implications for understanding the mechanisms of evolution of *M*. *tuberculosis* and is in contrast to what is observed during infection with the opportunistic pathogen *Burkholderia dolosa*^*6*^. After entering the airways of people with cystic fibrosis, *B*. *dolosa* establishes long-term colonization during which emergence of mutations in the population leads to diversification rather than genetic fixation, with potential for cooperative action among subclones. Because *M*. *tuberculosis* is a human obligate pathogen that has co-evolved for thousands of years with the same unique host species, the need for variation to establish successful infections in the lung may be reduced and could even be deleterious for the pathogen population [33].

Our results help explain the global phylogenetic structure of the MTBC. Although the MTBC is considered clonal, each lineage is distinct and associated with a specific human population[15], leading to the hypothesis that the genetic conservation of each lineage is the result of an enduring sympatric relationship with their host (same geographical origin). Several observational studies provide evidence supporting this model. Notably, a Swiss cohort study found that MTBC lineages tend to transmit preferentially among sympatric host populations[34]. Two other studies in Ghana offered a potential explanation for the geographical restriction of lineage 5 in West Africa, by showing association between these lineages and specific human ethnicities[35, 36]. A dominant lineage model of evolution can also help explain the various clinical outcomes resulting from infections with strains from distinct lineages of the MTBC. This would explain the finding that individuals from the Gambia that were infected with 'modern' lineages 2 and 4 were more likely to progress to active disease than individuals infected with 'ancient' lineage 6 which is endemic and likely evolved in that region[37].

The finding that within-host selection contributes to shape the *M*. *tuberculosis* genome is also supported by the increased diversity observed during *M*. *tuberculosis* growth in nutrient-rich culture media. For decades, laboratories have maintained *M*. *tuberculosis* H37Rv. Originally derived from a clinical strain (H37), it was recovered in 1905 from a patient with pulmonary tuberculosis. Early records showed the capacity of H37 to adapt to different in vitro conditions leading to phenotypic dissociation between virulent and avirulent derivatives[38]. Although the mechanisms by which this dissociation occurred were unknown, it highlighted the plasticity of *M*. *tuberculosis* metabolism and its central role for virulence. We found that in vitro culture conditions represent permissive environments for genetic drift and that these changes targeted enzymes involved in metabolic pathways dispensable during in vitro culture, but essential for optimal growth under nutrient-limiting conditions in vivo. These results demonstrate the capacity of *M*. *tuberculosis* to generate variation under permissive conditions.

A significant finding of this study is the impact of T cells in generating diversity in *M*. *tuberculosis* populations in vivo. By comparing genome sequences of *M*. *tuberculosis* populations isolated from WT and T cell deficient mice, we found that the presence of T cells was associated with the appearance of unique variants of the *M*. *tuberculosis* genome during prolonged *in vivo* infection. The findings reported here are in contrast with the hypothesis that T cells are strictly driving *M*. *tuberculosis* epitope conservation. Although the present results could either be due to direct recognition of specific peptide sequences by clonotypic T cells or to the indirect consequence of T cell activation and effector mechanisms, the possibility that direct recognition by T cells can contribute to sequence diversity is consistent with our recent finding that naturally occurring sequence variation in specific *M*. *tuberculosis* epitopes affects human T cell recognition[21]. In addition, the findings emphasize the point that the impact of T cell recognition, whether to promote conservation or diversity, is a function of the specific antigen/epitope and genetic locus, and that T cell recognition of distinct antigens can have different outcomes that may favor the pathogen or the host.

Overall, the results reported here reveal that purifying selection and increased genomic diversity are not two mutually exclusive processes during *M*. *tuberculosis* infection. While the impact of purifying selection was apparent shortly after the initial infection, increased genomic diversity occurred progressively and was observed after serial infection-transmission cycles over the course of months. Similar results were recently published that reveal evidence of both purifying selection and genome diversification in *M*. *tuberculosis* isolates obtained from distinct lesions and organs of HIV-coinfected humans that succumbed to infection[39]. Thus, in another context, prolonged infection leads to increased *M*. *tuberculosis* genetic diversity in humans despite overall purifying selection pressure. Together, the findings in immunocompetent humans, HIV-infected humans, and mice, all indicate that although selection favors overall sequence conservation in *M*. *tuberculosis*, there are also long term forces that favor diversifying selection, most likely to adapt to a new environment or to counter new stresses. Our findings will guide deeper investigation of the mechanisms used by *M*. *tuberculosis* to adapt and to continue to be a globally successful pathogen.

In conclusion, our findings indicate that *M*. *tuberculosis* genetic selection is driven by a deterministic process imposed by both genetic drift and within-host selection, leading to a dominant lineage mode of evolution. Although *M*. *tuberculosis* is not rapidly mutating, our results indicate that this pathogen is capable of genetic plasticity dictated by environmental changes. The necessity to adapt leads to selection and the contribution of dominant genotypes determined by the host. Our results also demonstrate for the first time the impact of T cells on sequence diversity of *M*. *tuberculosis* and indicates that T cell responses are a force that can promote diversity at specific sites rather than to only maintain conservation during infection.

## Materials and Methods

### Ethics statement

All animal experiments were done in accordance with procedures approved by the NYU School of Medicine Institutional Animal Care and Use Committee (IACUC - Laboratory Animal Care Protocol #160426–01). These IACUC regulations conformed to the national guidelines provided by the Guide for the Care and Use of Laboratory Animals of the National Institutes of Health.

### Mouse infection and sample collections

In an ABSL3 facility, *M*. *tuberculosis* H37Rv was cultured and used to infect 5 WT (C57BL/6) or 5 T cell-deficient (TCR β/δ^-/-^) mice by the aerosol route with ~100 CFU/ mouse. After 42 days of infection, mice were euthanized and their lungs homogenized in 5 ml of 7H9 culture medium and the bacterial population was allowed to expand during a minimal period (~1 week) in vitro. The culture from one mouse was then diluted to prepare an aerosol inoculum of 100 CFU, which was used to infect a new set of mice of the same background. At each passage, CFU were calculated and frozen stocks were made. Following the third (21 weeks) and sixth (42 weeks) mouse passages, a pool of *M*. *tuberculosis* from each mouse was used for DNA extraction and sequencing.

*M*. *bovis* BCG isolates from 4 patients were provided from the collection of the New York State Mycobacteriology Laboratory in the form of solid agar cultures. The samples were de-identified, but were known to have been obtained from 4 different adult patients treated for bladder cancer. For each culture, frozen stocks were made before entire plates were swept for gDNA preparation. The pharmaceutical grade *M*. *bovis* BCG Tice strain was obtained from Theracys.

### Illumina sequencing

Genomic DNA was extracted using a standard kit (Qiagen), and sequenced by GATC-Biotech. Illumina single read sequencing was performed with single-read of 51 bases and a target coverage of at least 3 million high-quality bases. On average, 9.5 million reads were obtained per isolate. We used Burrows-Wheeler Aligner (BWA)[40] to map the reads from the genome sequences against the H37Rv NC_000962 reference sequence. BWA outputs were analyzed and annotated using SAMtools[41], and ANNOVAR [42]. SNPs in genes annotated as PE/PPE genes, integrases, transposases, resolvases, maturases, or phages were removed from the analysis.

### Mutation identification, deep population sequencing

Bacteria from entire plates were pooled from each lung sample and sequenced with deep coverage. The reads were aligned to H37Rv NC_000962 reference sequence and we identified fixed mutations, appearing in all reads, and polymorphisms, appearing in only a fraction of the reads. For study of disseminated *M*. *bovis* BCG isolates, bacteria on entire plates were pooled and the sequence reads were aligned to the *M*. *bovis* BCG Tice reference sequence (SAMN03023974). To remove false positive polymorphic sites caused by systematic sequencing or alignment errors[43], we developed a set of thresholds and statistical tests that rejected polymorphic sites where the mutated and ancestral reads had significantly different properties. The population sequencing approach reliably detected polymorphisms where the minor allele frequency was greater than 10%, while decreasing the cost and labor required per sample. We considered a position to be polymorphic if it met the following quality thresholds in the given sample: *minor allele frequency*: more than 10% of reads supported a particular minor allele; *minor allele coverage*: at least 50 reads aligned in both the forward and reverse direction, and the total number of reads aligning is below the 99^th^ percentile of covered positions in that sample; *base quality*: average base quality (provided by sequencer) was greater than 20 for both the major and minor allele calls on both the forward and reverse strand; *mapping quality*: average mapping quality (provided by aligner) was greater than 19 for reads supporting both the major and minor alleles on both the forward and reverse strand; *indels*: no reads aligning to that position support an indel at any position along that read; *isogenic control*: More than 98.0% of reads aligning to this genomic position in the isogenic control support a major allele; *strand bias*: A *p-value* < 0.01 supporting a null hypothesis that the minor allele frequency for the SNPs identified in the mouse isolates is the same for reads aligning to both the forward and reverse strand (Fisher’s exact test)[6]. For some mutations identified in the inoculum population, the strand bias criterion was not taken into account if the polymorphism was confirmed after the 3^rd^ mouse passage and purified out after the 6^th^ mouse passage.

### Mutation confirmation

Minor variants identified at high frequency (>30%) in the bacterial populations were confirmed by Sanger sequencing of PCR amplification products using primers anchoring unique regions flanking the mutated genes. PCR was performed using the FastStart High fidelity PCR system (Roche). The purified product was diluted and submitted with the forward and reverse primers to Genewiz for dideoxy chain termination sequencing. BLAST was used to align the resulting sequences against the corresponding genes to confirm the presence of multiple peaks at the polymorphic positions.

### Characterization of protein functions and amino acid tolerance prediction

To characterize the families and functions of nSNP-encoding proteins, we used the following databases: Tuberculist (http://tuberculist.epfl.ch/), KEGG (http://www.genome.jp/kegg/), UniProt (http://www.uniprot.org/) and ModBase (http://modbase.compbio.ucsf.edu/modbase-cgi/index.cgi). To predict the impact of amino acid changes on protein function, we used 3 algorithms in parallel: Sift (http://sift.jcvi.org/), Polyphen-2 (http://genetics.bwh.harvard.edu/pph2/) and Provean (http://provean.jcvi.org/index.php). A mutations was considered deleterious if the outputs obtained from the 3 algorithms converged toward the same conclusion.

### Model description

According to the experimental results, there are two fixation states of polymorphisms for the frequency of mutated reads: one with zero representation (denoted as *F_0_*) and the other with full representation (denoted as *F_1_*). We introduced a variable y into the system to characterize the level of a decision marker that depicts the competitions between two fixation states. We assumed that *y* satisfies *y'* =*a* (1-*y*)-*b y*, where *a* represents the transition rate from *F_0_* to *F_1_*, and *b* represents the transition rate from *F_1_* to *F_0_*. Write y* = a/(a+b) and this quantity is indeed the equilibrium solution of the equation above. Furthermore, we assumed that the closer the value of y to y*, the more it favors *F_1_*. Meanwhile, the initial condition of *y was* set as follows: *y*(0) = 0 when the percentage of variant alleles in the inoculum population is below 50%, whereas *y*(0) = y* when the percentage of variant alleles in the inoculum population is above or equal to 50%. The percentage of *M*. *tuberculosis* variant alleles was defined as *x*. We assumed that *x* obeys the following dynamics: *x' = r(t)x(1-x)-d(t) x*, where the growth of x is assumed to be logistic, and *r(t)* and *d(t)* represent the time dependent per capita birth and death rates, respectively. Let *e_r_* and *e_d_* (or Δ*r* and Δ*d*) denote the baseline (or elevation of) birth and death rates of *x*, respectively. Let *T_d_* be the decision time. Then *r*(*t*) and *d*(*t*) can be written as: (1) *r* (*t*) = *e_r_+Δr*, if |y*-y(*T_d_*)|< *c and t> = T_d_*, and *r(t)= e_r_* otherwise; (2) *d*(*t*) = *e_d_+Δd*, if |y*-y(*T_d_*)|> = *c and t> = T_d_*, and *d(t) = e_d_* otherwise. Here c defines a prescribed decision threshold. Particularly, the fixation (achieved at the end) will be in favor of *F_1_* if the distance between the value of *y* and y* at the decision time is close enough and is less than c, and *F_0_* otherwise.

### Model fitting procedure

The proposed system of ordinary differential equations is analytically solvable. However, only the part of the system (i.e. *x)* is observable. Thus, the method of nonlinear least squares is not applicable to fit the model to the data. So, we estimated the model parameters from data by employing the general profiling procedure proposed by Ramsay et al.[29]. These parameter estimates along with estimated initial values of *x* component allowed us to solve the ordinary differential equations. We assumed a Gaussian error distribution with constant variance for all data points. This assumption was reasonable because the errors introduced during measurement of the data were not estimated explicitly and were likely to be the same for all data points. The general profiling procedure was implemented as two nested levels of optimization. In the first level, the solution (x(t),y(t)) of our model was approximated with smooth curves by penalized smoothing with ordinary differential equations defined penalty, conditional on the model parameter vector. In the second level, the model parameter vector is estimated by minimizing the weighted sum of square errors.

### Estimation of in vivo mutation rate from WGS data

Average mutation rate was estimated as in Ford, et al. 2011. In short, mutation rate (μ) was estimated using the equation[44]:

μ=m/[N×(t∕g)]

where the number of SNPs (m) was divide by the genome size (N = 4.4Mb) times the number of generations (t/g). We opted for a generation time of 20h for both mouse groups. M is defined by the number of SNPs observed, N is determined based on 93% coverage of a 4.4Mb genome (N = 4×10^6^), t is the total duration of each infection in hours, and g is the generation time in hours (20h).

## Supporting Information

S1 Table Frequencies and characteristics of polymorphic sites identified in the *M*. *tuberculosis* inoculum and mouse lung bacterial populations. (XLSX)

S2 Table Linkage inference using variation of single nucleotide polymorphism frequencies observed in the inoculum and mouse isolate populations. (XLSX)

S3 Table Characteristics of mutations identified in the *M*. *bovis* BCG populations isolated from human patients with disseminated infection. (XLSX)
